# Effectiveness of different types of hair traps for brown bear research and monitoring

**DOI:** 10.1371/journal.pone.0186605

**Published:** 2017-10-26

**Authors:** Teresa Berezowska-Cnota, Ignacio Luque-Márquez, Isabel Elguero-Claramunt, Katarzyna Bojarska, Henryk Okarma, Nuria Selva

**Affiliations:** 1 Institute of Nature Conservation, Polish Academy of Sciences, Krakow, Poland; 2 Institute of Environmental Sciences, Jagiellonian University, Krakow, Poland; Universidade de Aveiro, PORTUGAL

## Abstract

Non-invasive sampling by hair-trapping is increasingly used worldwide in wildlife research. Despite this rise and the potential of hair samples for ecology and conservation studies, the relative performance of hair collection devices has been rarely tested. Here, we compare the effectiveness of five types of hair traps for brown bears *Ursus arctos* in the Carpathian Mountains (SE Poland) and test the effects of trap type, season, number of days elapsed since trap installation and trap features on the trapping success in order to provide recommendations for optimal sampling in future studies. The trap types were corral, path-trap, “smola”(beechwood tar) tree-trap, turpentine tree-trap and natural rub. In 2010, we collected 858 hair samples during 2330 inspections of 175 hair traps and found that the most effective traps were smola tree-traps (mean percentage of successful inspections ± SD: 30.2% ± 26.0) and natural rubs (50.8% ± 16.7). Based on this finding, over the following 2 years we focused on 24 smola tree-traps and eight natural rubs. During this long-term survey (2010–2012, 969 inspections, 1322 samples collected) the trapping success increased with time and smola tree-traps achieved similar effectiveness to natural rubs (45.5% ± 29.7 and 45.9 ± 23.4, respectively). We show that when baiting smola tree-traps ten weeks prior to research or monitoring, sampling effectiveness can reach up to 30%. Taking into account the logistical and methodological constraints associated with detecting and using natural rubs for a proper survey design, we recommend using smola tree-traps baited in advance for hair sampling in wildlife studies.

## Introduction

Non-invasive sampling is nowadays commonly used worldwide in wildlife research (e.g. [1–3]). Its scope of potential applications is growing rapidly [4], as it allows to investigate animal populations without the need to physically capture or even directly observe the study species [5]. This is especially important when dealing with rare, elusive or endangered species [6], [7], such as the brown bear (*Ursus arctos*). Indeed, systematic and opportunistic collection of bear hair and scat samples has been widely used to estimate population size and density (e.g. [8–11]), assess genetic structure [12–14], measure population fragmentation [15], [16], identify concrete individuals that are of importance for management decisions [17–19], as well as to examine food habits [20–22], parasites [23], [24] and stress levels [25].

Among multiple non-invasive sampling methods, systematic hair-trapping seems to be especially acknowledged by bear specialists. Hair traps for bears were first designed in 1995 [26], and since then researchers have developed a wide variety of hair collection devices to sample ursids [27]. Although optimal hair collection devices vary among bear species [28], those constructed of barbed wire are most popular, because the wire facilitates hair collection, catches larger samples with more follicles, and reduces the percentage of mixed samples, i.e. containing hairs from various individuals [5]. Hairs can be passively left by bears on unbaited hair traps during the course of their normal activities, whereas baited methods need to evoke a certain behavioral response from an individual to collect a hair sample [27].

Despite the extensive use of non-invasive methods in bear research, little is known about the effectiveness of different bear hair collection devices. Although some studies have evaluated the accuracy and cost-effectiveness of different non-invasive sampling strategies for bears (e.g. [29], [10], [17]), none have assessed the comparative performance of different sampling devices. Here, we compared the effectiveness of five distinct types of hair traps generally used in bear research which apply both baited and passive approaches. We also examined factors influencing the trapping success, such as the type of trap, season, number of days elapsed since trap installation and trap features. The main goal of this study was to determine the most efficient brown bear hair collection device and to provide recommendations for optimal sampling in future studies.

## Materials and methods

### Ethics statement

The field study was done in strict accordance with legal requirements in Poland. It was conducted in the public forest lands, managed by the Polish State Forest Administration. Installation of hair traps do not require any permit in Poland. In spite of this, agreement from the Forest Districts in the study area was additionally guaranteed previous to the installation of hair-trapping stations. Brown bear hair samples in the present study were collected non-invasively. Therefore, sample collection did not require animal capture or handling. Necessary permissions to collect and store the samples at the Institute of Nature Conservation were obtained from the General Directorate for Environmental Protection in Poland.

### Study area

**Fig 1:**
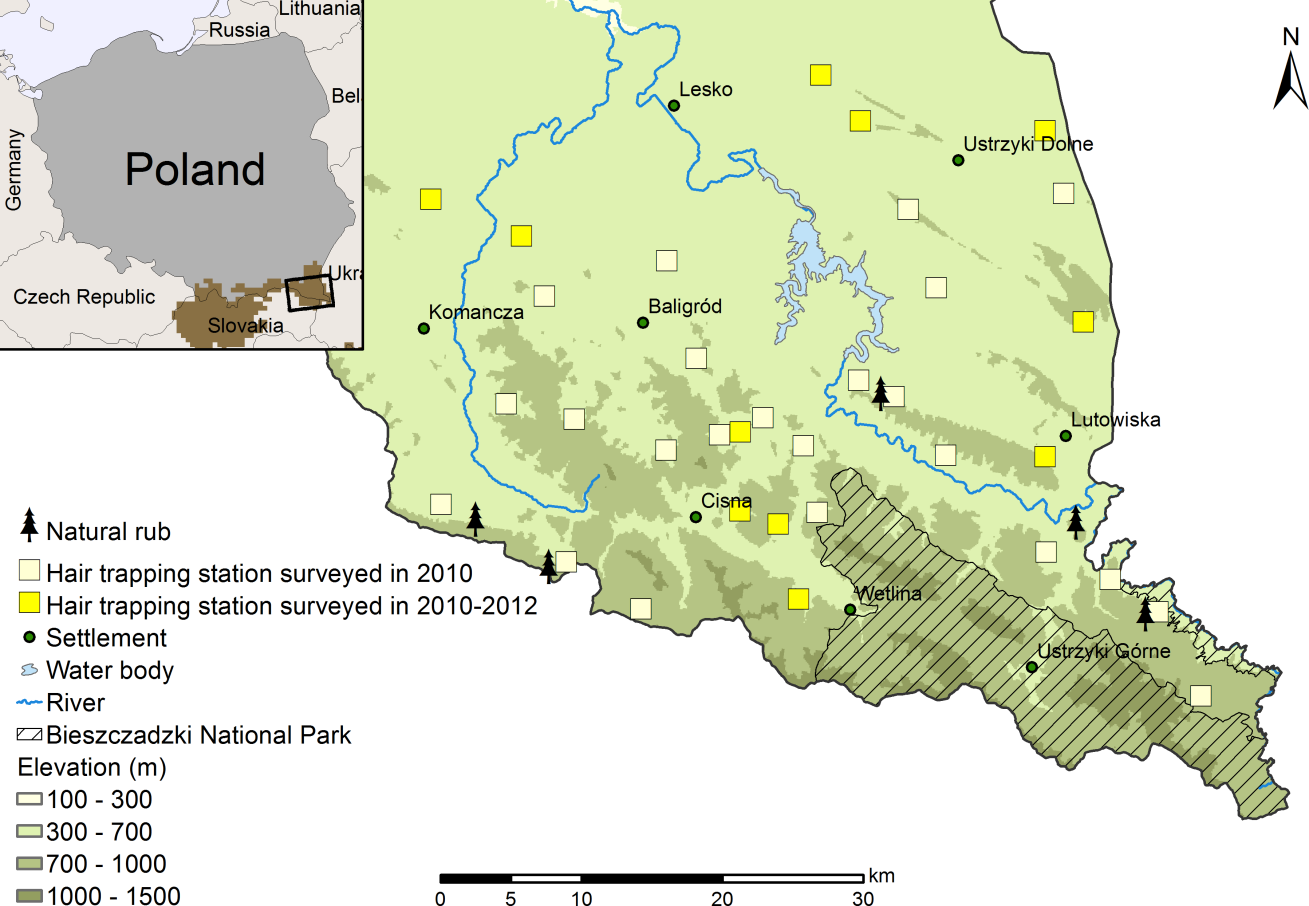
Study area and location of the hair-trapping stations. The study area in the Northeastern Carpathians (SE Poland) and the distribution of the 34 hair-trapping stations and five natural rub sites surveyed during the two phases of the study in 2010–2012. Hair-trapping stations consisted generally of four types of traps: one corral, two path-traps, one smola tree-trap and one turpentine tree-trap. Eight natural rubs found at five sites were included in the study. Distribution of brown bears in the Northeastern Carpathians [33] is shown on the map in the upper right corner.

The study was conducted in the Polish part of the Northeastern Carpathians, including the Bieszczady Mountains (Fig 1; N49°18′, E22°18′). The area is a mountain range with gentle slopes and altitudes varying from 420 to 1346 m a.s.l. [30]. Natural vegetation can be divided into three altitudinal zones: the foothill zone (<500 m a.s.l) which is nowadays mostly occupied by human settlements and agriculture, with a limited cover of mixed deciduous forests; the lower montane zone (500–1150 m a.s.l.) primarily consisting of forests dominated by beech (*Fagus sylvatica*) and silver fir (*Abies alba*); the zone above the upper tree line (called “polonina”, >1150 m a.s.l.), where subalpine and alpine communities are typical [31], [32]. This region has a moderately cool and humid climate with marked continental influence. The mean annual air temperature during the study period (2010–2012) ranged from 7.1 to 7.5°C and the average annual precipitation fluctuated between 821–1188 mm (data provided by the Polish Institute of Meteorology and Water Management—National Research Institute).

The vertebrate community of the study area is very rich. Large carnivores are represented by the brown bear, the Eurasian lynx (*Lynx lynx*) and the wolf (*Canis lupus*). It is one of the main strongholds for the brown bear in Poland, hosting about 55 individuals [34]. Bears living in the study area are mostly transboundary and belong to the Carpathian population, which currently extends over Czech Republic, Slovakia, Poland, Ukraine, Hungary, Romania and Serbia [35]. Excepting the protected area of the Bieszczady National Park (ca. 300 km^2^), the area undergoes timber harvest and intensive game management. Our study area covers approximately 1300 km^2^ of this commercially exploited forest, where a total of 212 ungulate feeding sites have been inventoried. The brown bear is a strictly protected species in Poland [34].

### Hair-trapping surveys

A total of 34 fixed hair-trapping stations were installed in the study area in January-February 2010 (except three stations in April and June). Hair-trapping stations were distributed in a regular pattern across the study area (Fig 1); the mean nearest neighbor distance among stations was 4.8 km (SD ± 1.8). In order to maximize capture probabilities, we located each trapping station in the vicinity of areas frequently used by brown bears, such as ungulate feeding sites [36]. Additionally, trees used naturally by bears for rubbing (hereafter called natural rubs) detected in the area were also included in the study for a comparison.

**Fig 2:**
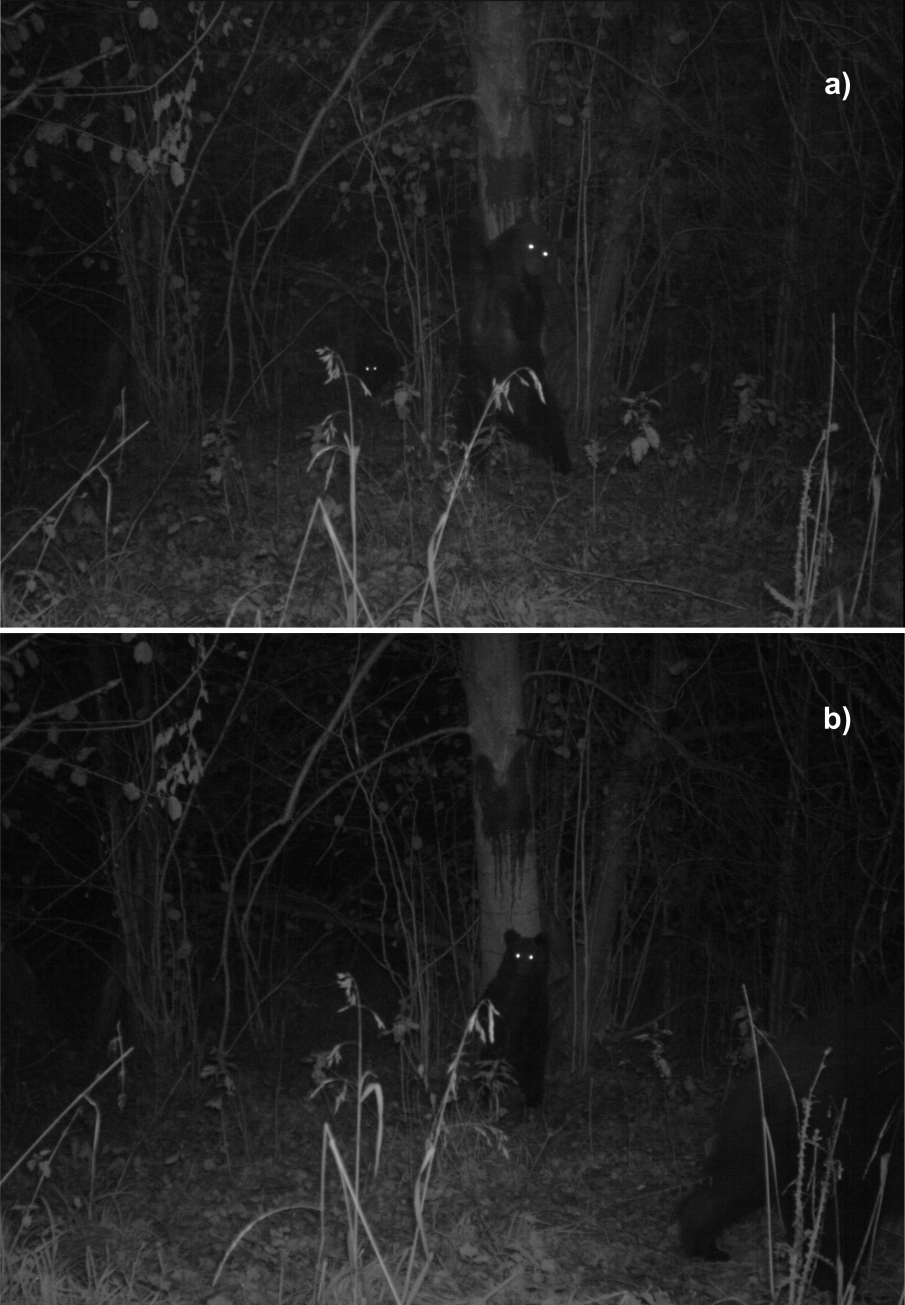
A brown bear family photo-trapped at a hair-trapping station in the Northeastern Carpathians (SE Poland) while rubbing against a smola tree-trap: (a) female and (b) young.

Each hair-trapping station consisted generally of four different types of hair traps: one corral, two path-traps, one tree-trap baited with beechwood tar (“smoła” in Polish, “smola” hereafter, Fig 2) and one tree-trap baited with turpentine (S1 Table). Hair corrals were constructed following Woods et al. [26] and Kendall and McKelvey [27]. A single strand of barbed wire was stretched around four or more trees at a height of 50–60 cm, forming a polygon with sides of about two meters each, and enclosing a pile of branches and woody debris in the center. The pile was treated with approximately 300 ml of inedible liquid mixture of cattle blood and rotten fish juice in a ratio of 3:1, similar to Kendall et al. [12]. Fencing staples were used to secure wire to trees. The path-traps consisted of a barbed wire strand strung across an animal trail at a height of 50–60 cm. It was a passive trap, without any attractant. Tree-traps comprised three strands of barbed wire of approximately 50 cm attached horizontally to the tree trunk and separated about 50 cm apart; the lowest strand was fixed 50–60 cm from the ground. Tree-traps were baited with two handfuls (about 150 ml) of a distinct lure, turpentine or smola, to induce bear rubbing. We chose coniferous trees (fir, larch *Larix decidua*, spruce *Picea abies* and Scots pine *Pinus sylvestris*) located on forest edges or in other conspicuous locations according to bear’s rubbing preferences reported in previous studies [37]–[39]. Natural rubs were fitted with barbed wire, following the same procedures as with tree-traps, but they remained unbaited.

Hair traps were systematically surveyed from March to November, i.e. during the period of high bear activity in the area. In winter (December-February), due to low bear activity and harsh field conditions, we controlled only traps at sites that were accessible to researchers. The study consisted of two phases:

- All-traps survey: in 2010 (from March to December) we inspected the hair-trapping stations (n = 34) and natural rubs (n = 8) over multiple sampling sessions at approximately 14-day intervals in order to assess the effectiveness of the five types of hair traps.
- Rub-trees survey: based on the results from the all-traps survey, we focused on a subsample of trees (n = 32), including eight natural rubs and 24 smola tree-traps (rub-trees hereafter). These trees were surveyed then at larger time intervals (once per month) in 2011 and 2012.

During trap inspections hairs were identified macroscopically by experienced field staff. Each bunch of bear hairs found on a single wire barb was considered as one sample, regardless of the number of hairs trapped. Subsequently, we collected all remaining hairs from the wire strands, and also from the tree bark in the case of tree-traps, and labeled them as a single sample. After that, the wires and tree bark were flamed to ensure that no hairs remain and that new hairs will be distinguishable in the next inspection. In the case of baited traps, scent lure was refreshed. Traps were repaired during the inspections if needed. Hair samples were stored in dry labeled envelopes at room temperature with access to fresh air. Uncertain bear hair samples were later identified using reference material and disregarded if they did not belong to brown bears.

We compiled a database that contains the entire survey history of each hair trap, and included for each inspection the date, the trapping success (i.e. whether bear hairs were collected or not, 1/0) and the total number of samples collected. We noted the GPS coordinates of each hair-trapping station and natural rubs. For the rub-trees survey, we recorded the tree species and the diameter at breast height (cm) of both natural rubs and smola tree-traps.

### Data analysis

#### All-traps survey

We used field data obtained in 2010 to investigate differences in the effectiveness among five types of hair traps: corral, path-trap, smola tree-trap, turpentine tree-trap and natural rub. Sampling effectiveness of each hair trap was evaluated based on the trapping success of all inspections and was calculated as percentage of positive inspections (i.e. those with bear hair samples collected) out of the total number of inspections. Those hair-trapping stations where no sample was found at any trap during the whole survey (e.g. because of no or low presence of bears in the area) were excluded from the analysis. Records obtained during inspections of destroyed and, thus, not fully functioning hair traps were also excluded from the analysis.

We used a generalized linear mixed model (GLMM) to relate trapping success (1/0) to the type of hair trap and bear activity season. Bear activity was classified according to the species’ phenological periods or physiological states instead of calendar months. We divided the year into four seasons: winter dormancy (December-February), hypophagia (March-April), mating season (May-June) and hyperphagia (July-November). Each season was coded as an integer according to the level of general bear activity as follows: 1- wintering; 2- hypophagia; 3- hyperphagia; 4- mating. We included the location code of hair-trapping stations and natural rubs as a random effect in the model.

#### Rub-trees survey

We merged the data obtained for a subsample of rub-trees (24 smola tree-traps and eight natural rubs, which were the most effective trap types) surveyed during the second phase of the study (2011–2012) with the data collected in 2010 for the same rub-trees. We then calculated the effectiveness of rub-trees to assess their performance over a longer time period (2010–2012). We performed a GLMM to test the effects on trapping success of tree diameter (scaled), tree species, bear activity season as described above and the cumulative number of days (log-transformed) elapsed since the installation of a given trap. Tree ID was fitted as a random term.

We used an information-theoretic approach for model selection [40] and examined a set of a priori specified models for each analysis (all-traps and rub-trees). We run a full model incorporating all explanatory variables and ranked the resulting set of candidate models according to the Akaike Information Criterion (AIC; S2 Table). Additionally, we run the selected model for each type of rub-tree (smola tree-traps and natural rubs) separately to predict the probability of hair-trapping and to provide recommendations for future studies. All models were fitted in R (version 3.0.3), [41] with the lme4 package [42] (function glmer) using a logit link function and a binomial error distribution. The package MuMIn was used for model selection [43].

## Results

### All-traps survey

**Fig 3:**
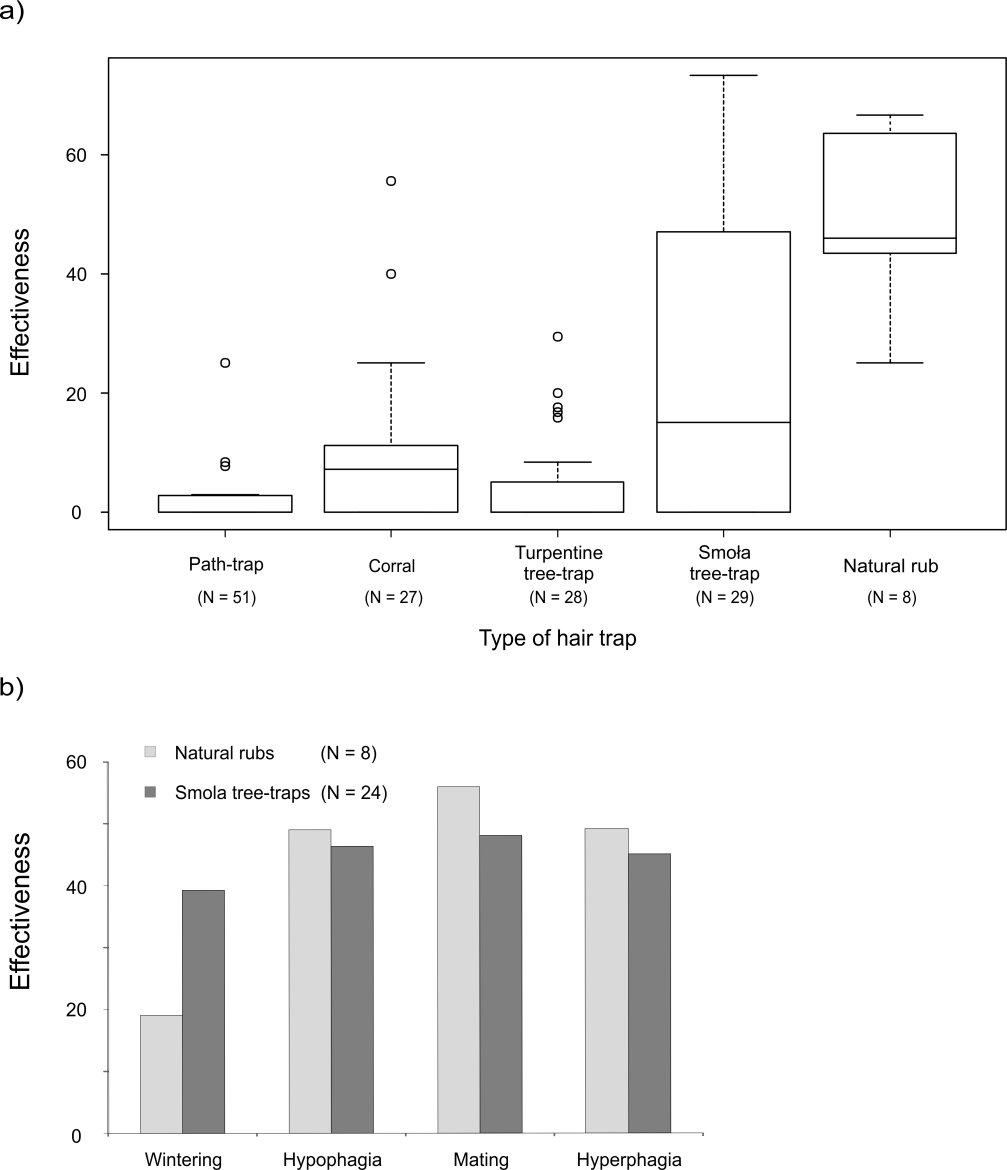
The effectiveness of hair traps, calculated as the percentage of inspections with bear hair samples out of the total number of inspections. (a) Results of the all-traps survey (2010) showing the effectiveness of different types of hair traps: path-trap, corral, turpentine tree-trap, smola tree-trap and natural rub; (b) Results of the rub-trees survey (2010–2012) showing the effectiveness of natural rubs and smola tree-traps in relation to bear activity seasons: wintering (December-February), hypophagia (March-April), mating season (May-June) and hyperphagia (July-November).

During the first phase of the survey in 2010 we conducted a total of 2831 inspections of 175 hair traps. Eighteen per cent of the inspections corresponded to seven negative hair-trapping stations–containing 32 individual traps–which did not collect any bear hair, and were excluded from the analysis. Therefore, for calculations of trap effectiveness we considered 2330 records obtained during inspections of 27 hair-trapping stations and eight natural rubs. The mean number of inspections (± SD) per hair trap was 16.3 (± 3.0). Overall, we collected 858 bear hair samples from 76 hair traps. The effectiveness of different types of hair traps showed a pronounced variation. Most effective were natural rubs (mean ± SD: 50.8% ± 16.7), followed by smola tree-traps (30.2% ± 26.0). Both were distinctly more successful than the other trap devices (Fig 3). The mean number of samples collected per positive inspection varied among trap types, ranging from 1.5 for path-traps to 3.3 for smola tree-traps (Table 1).

**Table 1:** Summary of the brown bear hair-trap surveys for 27 positive hair-trapping stations and eight natural rubs in 2010 and 32 rub-trees in 2010–2012.

Type of hair trap	No. traps	Positive traps^a^	No. inspections	Effectiveness^b^^,^^c^	No. samples collected	No. samples / positive inspection^c^	Max no. samples collected
**All-traps survey (2010)**							
Corral	27	85.2	457	12.4 ± 12.2	135	2.4 ± 2.6	12
Path-trap	51	23.5	826	2.3 ± 5.2	30	1.5 ± 0.9	4
Turpentine tree-trap	28	39.3	477	4.8 ± 8.0	66	2.4 ± 2.0	8
Smola tree-trap	29	75.9	443	30.2 ± 26.0	462	3.3 ± 2.6	13
Natural rub	8	100.0	127	50.8 ± 16.7	165	2.6 ± 2.2	9
**Rub-trees survey (2010–2012)**							
Smola tree-trap	24	100.0	728	45.5 ± 29.7	1025	3.1 ± 2.4	13
Natural rub	8	100.0	241	45.9 ± 23.4	297	2.6 ± 2.1	9

^a^percentage of traps with bear hair samples out of the total number of traps of a given type

^b^percentage of inspections with bear hair samples out of the total number of inspections

^c^mean ± SE

The GLMM showed that the type of hair trap was the main factor affecting trapping success; natural rubs had the highest probability of hair-trapping, followed by smola tree-trap. The other types of traps were significantly less effective (Table 2). Bear activity season did not have a significant effect on trapping success during this survey.

**Table 2:** Factors affecting hair-trapping success.

	Fixed effect	Estimate	SE	*P*
All-traps	Intercept^a^	-0.233	0.357	0.514
(2010)	Corral	-2.07	0.358	<0.001
	Path-trap	-3.85	0.399	<0.001
	Smola tree-trap	-0.681	0.343	0.0469
	Turpentine tree-trap	-3.05	0.387	<0.001
Rub-trees	Intercept	-3.23	0.557	<0.001
(2010–2012)	Days elapsed since trap installation	0.417	0.0808	<0.001
	Activity^b^	0.248	0.0803	0.00201
a. Smola tree-traps	Intercept	-4.49	0.711	<0.001
	Days elapsed since trap installation	0.651	0.102	<0.001
	Activity^b^	0.227	0.096	0.0177
b. Natural rubs	Intercept	-0.493	0.923	0.593
	Days elapsed since trap installation	-0.140	0.144	0.331
	Activity^b^	0.402	0.159	0.0116

^a^natural rub included as intercept

^b^bear activity season, coded as an integer according to the level of bear activity

1- wintering; 2- hypophagia; 3- hyperphagia; 4- mating

Results of the most parsimonious Generalized Linear Mixed Models explaining the variation in the probability of bear hair-trapping (1/0) for all traps (survey in 2010) and for the rub-trees (survey 2010–2012) in the Northeastern Carpathians. The rub-trees included the most effective hair traps (natural rubs and smola tree-traps); the model for the rub-trees survey was also run separately for each type of rub-tree. All models were fitted using a logit link function and a binomial error distribution (package lme4, R version 3.0.3).

### Rub-trees survey

From a total of 969 inspections of rub-trees (n = 32) in 2010–2012, 46% were successful, i.e. at least one bear hair sample was collected. During this long-term survey smola tree-traps achieved similar effectiveness to natural rubs (Table 1). Rub-trees effectiveness differed slightly among bear activity seasons (Fig 3). In total, we collected 1322 bear hair samples. Both types of rub-trees provided a similar mean number of hair samples during positive inspections (Table 1).

**Fig 4:**
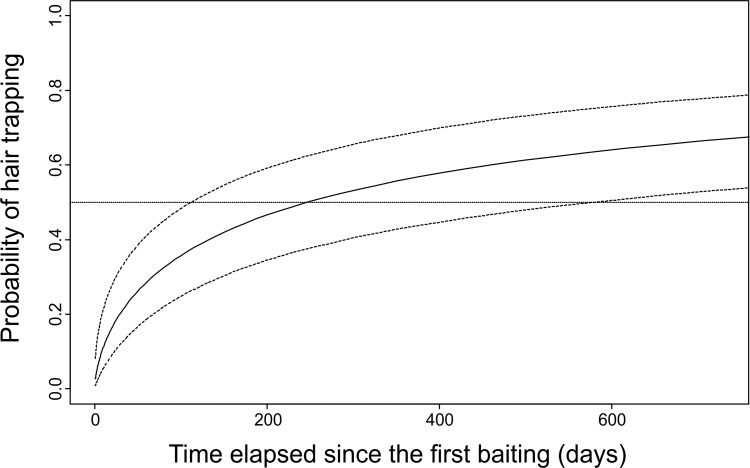
Predicted probability of sampling bear hairs at smola tree-traps in relation to the time elapsed since the first baiting. Estimates of the probability of hair-trapping with 95% confidence intervals (dashed lines) are generated from the Generalized Linear Mixed Model (logit link function and a binomial error distribution) as a function of the bear activity season and time elapsed since trap installation. Tree ID was set as a random effect. The predictions of the sampling effectiveness of the smola tree-trap were made for the mating season.

The GLMM indicated that the number of days elapsed since trap installation had the strongest effect on trapping success of rub-trees (Table 2). Bear activity season also had an influence; mating season was the time with highest success in hair-trapping for rub-trees. Tree diameter (range: 50–211 cm) and species (fir: 12% of the rub-trees, larch: 16%, spruce: 31%, Scots pine: 41%) did not affect hair-trapping success. When running the selected model separately for each type of rub-tree, we found that the time elapsed since trap installation explained most of the variation for smola tree-traps. However, this factor was not relevant for the trapping success of natural rubs, but season was important (Table 2, Fig 3). Model predictions showed that after 10 weeks of baiting, smola tree-traps can reach up to 30% of trapping success (Fig 4).

## Discussion

Given the increasing use of non-invasive sampling in wildlife studies, it is crucial to explicitly evaluate the effectiveness of different methods of sample collection for a successful study design. Our results clearly show that (1) the success of non-invasive studies involving hair-trapping strongly depends on the type of traps used; (2) proper sampling devices and attractants can be nearly as efficient as natural rubs in the case of bears; (3) it is important to bait the traps in advance to increase sampling effectiveness; and, (4) traps’ effectiveness can be additionally maximized by sampling in certain seasons, like the mating time. These findings can help to reduce logistical and financial efforts needed to monitor bears and other elusive wildlife in large study areas.

To our knowledge, this is the first study testing the effectiveness of multiple bear hair sampling devices, which were working simultaneously at selected sites, and, thus, allowed for a comparison. Although the use of different types of hair traps in bear studies is not rare, researchers mostly report combined data for all types of traps, as often the assessment is done from the perspective of genetic research, but not of the effectiveness of the trap itself (e.g. [14]). Corrals, which generally produce higher detection rates in comparison to rub objects [44], [45] and are the most popular type of bear hair traps used in North America (e.g. [46], [29]), had four times lower effectiveness than natural rubs in our survey. In Eurasia, hair traps utilizing rubbing behavior seem to be more effective than corrals [28] and therefore, more widely used by researchers in field studies. The ultimate reason is unknown, but it may be related to the fact that, contrary to North America, supplementary feeding practices are common in Europe and occur in six of the ten European bear populations [35]. This may make bears less interested in food attractants, as those used in corrals. Moreover, bears tend to rub on trees in regularly visited areas, such as ungulate feeding sites in our area [36], where the need to defend food and/or mates resources is elevated [47], [48]. Path-traps were most ineffective and are used rather rarely in bear studies, except in areas where bears aggregate, for instance near salmon spawning streams [49].

Natural rubs have been commonly used as hair traps [44], [5], [28], [45]. Here, we introduce a modification of the trapping device, which consists of “imitating” a natural rub by enticing bears to rub on tree-traps scented with smola. Rubbing against strongly smelling substances is a common behavior among mammals [47]. It has been reported that animal’s scent choice can affect survey results [50], [51], because carnivores display differential rubbing responses to distinct lures [52]. The success of smola tree-traps can be explained by the strong smell of smola, a thick, oily liquid obtained during dry distillation of beech wood, and by the fact that this dense substance remains on the bark of baited trees for a very long time, even after rainstorms. This is consistent with the study conducted in Greece using power poles by Karamanlidis et al. [53], who showed that creosote, also a tar distillation product used as a preservative for wood, is effective in stimulating bear rubbing. Although it is suggested that the strong smell of resin may trigger bear rubbing behavior [39], turpentine tree-traps were not as effective as smola ones, probably because turpentine evaporates quickly when exposed to air.

Some studies show that bears tend to select coniferous trees for rubbing, mainly fir and spruce [38], [39], and with relatively large diameters [37], [48], [39]. In our study we only used coniferous trees and we did not observe that tree-trap effectiveness was affected by the tree species or the diameter. When selecting trees for hair traps it may be more important to look for trees with long, easily accessible trunks and without lower branches. Additionally, we showed that both, smola tree-traps and natural rubs, had the highest effectiveness in the mating season (May-June). Intensified rubbing activity of bears and utilization of rub objects during mating time has been demonstrated also for bears living in other parts of the world [37], [53], [39]. Although sampling rub-trees in the mating season can maximize effectiveness, researchers should be aware of the risk of male-biased detection, particularly during this period (e.g. [53], [45]).

The duration of baiting is pivotal for sampling effectiveness, as bears may need some time to encounter a trap and rub and/or find the attractant. In the case of traps baited with food, there is a risk that some bears develop a trap-happy response, which can result in higher recapture rates of those individuals. But this is observed only when the bait consists of consumable food and the animal receives a food reward as a consequence of using the trap. Attractants lacking a food reward may have an opposite effect: once the animal discovers that there is no food, it might not be interested in revisiting the trap [54], [27]. In that case, an increasing effectiveness with time would not be observed, as it was the case of smola tree-traps in our study. Recent research suggests that tree-rubbing serves as chemical marking for intraspecific communication [38], [48], [39]. Our results support that bear tree rubbing enhances rubbing behavior by other individuals, as the effectiveness of rub-trees used by bears increased with time. Additionally, in 2014, we inspected the trees that were the smola tree-traps in this study and found that more than 66% were still rubbed by bears, in spite of not being baited for more than 2 years, since our survey finished. Those trees have become natural rubs. This has important implications for wildlife managers as it can improve the cost-effectiveness of long-term monitoring and periodical studies of brown bears and other wildlife.

The effectiveness of hair traps may be influenced by various factors besides those included in this study, such as weather conditions, local bear density, individual behavior in relation to traps, the level of human disturbance, proximity to bear movement paths or presence of abundant food resources, like large carcasses or feeding sites (e.g. [27], [55], [45]). If hair sampling is conducted for the purposes of genetic analysis, investigators should be especially aware of the effects of temperature and rainfall [27], [55], [45], and adjust the periods between trap inspections to local weather conditions. Our recommendations refer to studies conducted in temperate climates. For other regions, we suggest to first conduct pilot studies to test the effectiveness of different types of traps at the same sites and optimize the survey design in subsequent study periods accordingly.

Our results demonstrate the effectiveness of smola tree-traps as trapping devices to sample brown bear hairs. Baiting smola tree-traps in advance significantly increases sampling effectiveness (10 weeks before for 30% trap effectiveness). Taking into account that in long-term studies smola tree-traps can achieve a similar sampling effectiveness to natural rubs, and the difficulties associated with finding natural rubs in the field, we strongly recommend the use of smola tree-traps to collect bear hair samples in future studies.

## Supporting information

S1 Dataset Hair-trapping data. (XLSX)

S1 Table Results of the survey of five types of hair traps (corral, path-trap, smola tree-trap, turpentine tree-trap, natural rub), conducted from March to December 2010 in the Northeastern Carpathians, SE Poland, showing the number of traps, total number of field inspections, number of positive inspections (i.e. with at least one sample collected) and the effectiveness of a given type of hair trap at each of the 27 positive hair-trapping stations and five sites with natural rubs. Negative hair-trapping stations are not included in the table.
(PDF)

S2 Table Summary of model selection explaining the variation in the probability of bear hair-trapping (1/0) in relation to the type of trap (Type), bear activity season (Activity), the time elapsed since trap installation (Days), the tree diameter at breast height (DBH), and tree species (Species) for all-traps surveyed in 2010 and rub-trees surveyed in 2010–2012 in the Northeastern Carpathians. The location code of hair-trapping stations and natural rubs (for all-traps survey) and tree ID (for rub-trees survey) were included as random factors. All GLMMs were fitted using a logit link function and a binomial error distribution (package lme4, R version 3.0.3).
(PDF)
