# Supplementary material for: Effectiveness of different types of hair traps for brown bear research and monitoring
Source: PLoS One. 2017 Oct 26;12(10):e0186605. doi: 10.1371/journal.pone.0186605 (PMC5657975; doi:10.1371/journal.pone.0186605)
Supplement: S1 Table — Negative hair-trapping stations are not included in the table. (PDF) [file pone.0186605.s002.pdf]

S1 Table. Results of the survey of five types of hair traps (corral, path-trap, smola tree-trap, turpentine tree-trap, natural rub), conducted from March to December 2010 in the Northeastern Carpathians, SE Poland, showing the number of traps, total number of field inspections, number of positive inspections (i.e. with at least one sample collected) and the effectiveness of a given type of hair trap at each of the 27 positive hair-trapping stations and five sites with natural rubs. Negative hair-trapping stations are not included in the table.

	CORRAL				PATH-TRAP				SMOLA TREE-TRAP				TURPENTINE TREE-TRAP				NATURAL RUB			
LOCATION	No. hair traps	No. inspections	No. positive inspections	Effectiveness of hair traps*	No. hair traps	No. inspections	No. positive inspections	Effectiveness of hair traps*	No. hair traps	No. inspections	No. positive inspections	Effectiveness of hair traps*	No. hair traps	No. inspections	No. positive inspections	Effectiveness of hair traps*	No. hair traps	No. inspections	No. positive inspections	Effectiveness of hair traps*
Balnica	1	18	2	11.1	2	36	1	2.8	1	17	11	64.7	1	18	0	0.0	1	16	4	25.0
Bandrow	1	15	3	20.0	2	31	0	0.0	1	15	7	46.7	1	17	0	0.0				
Bystre	1	17	0	0.0	2	34	1	2.9	1	16	0	0.0	1	17	0	0.0				
Jablonki	1	17	2	11.8	2	32	0	0.0	1	15	7	46.7	1	17	0	0.0				
Jarzabek	1	18	1	5.6	2	36	0	0.0	1	16	0	0.0	1	18	0	0.0				
Jaworzec	1	19	2	10.5	2	38	0	0.0	1	17	3	17.6	1	19	3	15.8				
Kalnica	1	18	2	11.1	2	28	0	0.0	1	16	0	0.0	1	17	0	0.0				
Kolonice	1	18	1	5.6	2	36	1	2.8	2	21	14	66.7	1	20	1	5.0				
Lopienka	1	6	1	16.7	1	4	0	0.0	1	4	0	0.0	1	6	0	0.0				
Lopienka2	1	15	6	40.0					1	15	11	73.3	1	15	3	20.0				
Mikow	1	19	1	5.3	2	38	1	2.6	1	19	1	5.3	1	20	1	5.0				
Muczne	1	16	4	25.0	2	31	0	0.0	1	14	3	21.4	1	16	0	0.0				
Muczne2	1	12	1	8.3	2	24	2	8.3	1	12	2	16.7	1	12	1	8.3	1	11	7	63.6
Olchowiec	1	17	0	0.0	2	18	0	0.0	2	33	11	33.3	1	18	0	0.0				
Polanki	1	18	2	11.1	2	26	2	7.7	1	16	8	50.0	2	34	10	29.4				
Roztoki	1	19	1	5.3	2	35	0	0.0	1	18	6	33.3	1	19	0	0.0				
Sekowiec	1	18	2	11.1	2	36	0	0.0	1	17	11	64.7	1	18	0	0.0				
Smerek	1	17	1	5.9	2	29	0	0.0	1	16	0	0.0	1	17	1	5.9				
Steznica	1	14	0	0.0	2	30	0	0.0	1	15	2	13.3	1	17	0	0.0				
Stuposiany	1	19	4	21.1	2	38	1	2.6	1	17	10	58.8	1	19	0	0.0				
Stuposiany2																	3	48	22	45.8
Tarnawa	1	18	10	55.6	2	36	9	25.0	1	16	11	68.8	1	18	3	16.7				
Telesnica	1	18	1	5.6	2	34	1	2.9	1	16	0	0.0	1	18	0	0.0				
W. Michowa																	2	34	18	52.9
Zawoj	1	19	2	10.5	2	36	1	2.8	1	17	2	11.8	1	17	3	17.6				
Zernica	1	18	0	0.0	2	34	0	0.0	1	16	8	50.0	1	18	0	0.0				
Zubensko	1	18	2	11.1	2	36	0	0.0	1	17	8	47.1	1	18	0	0.0				
Zubrze	1	18	3	16.7	2	36	0	0.0	1	16	4	25.0	1	16	1	6.3	1	18	12	66.7
Zukow	1	18	2	11.1	2	34	0	0.0	1	16	0	0.0	1	18	0	0.0				

*Calculated as a percentage of positive inspections from the total number of inspections
