# Supplementary material for: Effectiveness of different types of hair traps for brown bear research and monitoring
Source: PLoS One. 2017 Oct 26;12(10):e0186605. doi: 10.1371/journal.pone.0186605 (PMC5657975; doi:10.1371/journal.pone.0186605)
Supplement: S2 Table — The location code of hair-trapping stations and natural rubs (for all-traps survey) and tree ID (for rub-trees survey) were included as random factors. All GLMMs were fitted using a logit link function and a binomial error distribution (package lme4, R version 3.0.3). (PDF) [file pone.0186605.s003.pdf]

S2 Table. Summary of model selection explaining the variation in the probability of bear hair-trapping (1/0) in relation to the type of trap (Type), bear activity season (Activity), the time elapsed since trap installation (Days), the tree diameter at breast height (DBH), and tree species (Species) for all-traps surveyed in 2010 and rub-trees surveyed in 2010-2012 in the Northeastern Carpathians. The location code of hair-trapping stations and natural rubs (for all-traps survey) and tree ID (for rub-trees survey) were included as random factors. All GLMMs were fitted using a logit link function and a binomial error distribution (package lme4, R version 3.0.3).

Variables	logLik	AIC	ΔAIC	w_i	df
Response: Success in hair-trapping					
**All-traps survey (2010)**					
Type + (1| Station)	-679.9	1371.8	0.00	0.700	6
Type + Activity + (1| Station)	-679.7	1373.5	1.70	0.300	7
(1| Station)	-828.3	1660.6	288.81	0.000	2
Activity + (1| Station)	-828.3	1662.6	290.74	0.000	3
**Rub-trees survey (2010-2012)**					
Activity + Days + (1| Tree ID)	-610.4	1228.9	0.00	0.430	4
Activity + Days + DBH + (1| Tree ID)	-610.2	1230.5	1.58	0.195	5
Activity + Days + Type + (1| Tree ID)	-610.3	1230.7	1.83	0.172	5
Activity + Days + DBH + Type + (1| Tree ID)	-610.1	1232.4	3.45	0.077	6
Activity + Days + Species + (1| Tree ID)	-609.4	1233.0	4.10	0.055	7
Activity + Days + DBH + Species + (1| Tree ID)	-609.4	1234.9	5.98	0.022	8
Activity + Days + Species + Type + (1| Tree ID)	-609.4	1235.0	6.12	0.020	8
Days + (1| Tree ID)	-615.3	1236.5	7.63	0.009	3
Activity + Days + DBH + Species + Type + (1| Tree ID)	-609.4	1236.9	8.00	0.008	9
Days + DBH + (1| Tree ID)	-615.1	1238.2	9.30	0.004	4
Days + Type + (1| Tree ID)	-615.2	1238.4	9.46	0.004	4
Days + DBH + Type + (1| Tree ID)	-615.0	1240.1	11.2	0.002	5
Days + Species + (1| Tree ID)	-614.4	1240.9	12.0	0.001	6
Days + DBH + Species + (1| Tree ID)	-614.3	1242.8	13.9	0.000	7
Days + Species + Type + (1| Tree ID)	-614.4	1242.9	14.0	0.000	7
Days + DBH + Species + Type + (1| Tree ID)	-614.3	1244.8	15.9	0.000	8
Activity + (1| Tree ID)	-624.6	1255.2	26.3	0.000	3
Activity + DBH + (1| Tree ID)	-624.4	1256.8	27.8	0.000	4
Activity + Type + (1| Tree ID)	-624.5	1257.0	28.1	0.000	4
Activity + DBH + Type + (1| Tree ID)	-624.3	1258.6	29.7	0.000	5
Activity + Species + (1| Tree ID)	-623.5	1259.1	30.2	0.000	6
(1| Tree ID)	-627.7	1259.4	30.5	0.000	2
Activity + DBH + Species + (1| Tree ID)	-623.4	1260.9	32.0	0.000	7
DBH + (1| Tree ID)	-627.5	1261.0	32.1	0.000	3
Activity + Species + Type + (1| Tree ID)	-623.5	1261.1	32.2	0.000	7
Type + (1| Tree ID)	-627.6	1261.2	32.3	0.000	3
DBH + Type + (1| Tree ID)	-627.4	1262.8	33.9	0.000	4
Activity + DBH + Species + Type + (1| Tree ID)	-623.4	1262.9	34.0	0.000	8
Species + (1| Tree ID)	-626.7	1263.5	34.6	0.000	5
DBH + Species + (1| Tree ID)	-626.6	1265.4	36.5	0.000	6
Species + Type + (1| Tree ID)	-626.7	1265.5	36.6	0.000	6
DBH + Species + Type + (1| Tree ID)	-626.6	1267.4	38.5	0.000	7

Activity = coded as an integer according to the level of bear activity (1- wintering; 2- hypophagia; 3- hyperphagia; 4- mating)

Days = time elapsed since trap installation (number of days, log-transformed)

DBH = tree diameter at breast height (DBH, scaled)

Species = tree species (fir, larch, spruce, Scots pine)

Type = type of hair trap (natural rub, corral, path-trap, smola tree-trap, turpentine tree-trap)
